# Hidradenitis Suppurativa: Current Understanding of Pathogenic Mechanisms and Suggestion for Treatment Algorithm

**DOI:** 10.3389/fmed.2020.00068

**Published:** 2020-03-04

**Authors:** S. Morteza Seyed Jafari, Robert E. Hunger, Christoph Schlapbach

**Affiliations:** Department of Dermatology, Inselspital, Bern University Hospital, Bern, Switzerland

**Keywords:** hidradenitis suppurativa, pathomechanism, pathogenesis, therapy, treatment

## Abstract

Hidradenitis suppurativa is one of the most distressing dermatological conditions and has a significant negative impact on patients' quality of life. However, the exact pathogenic mechanisms remain incompletely understood and—therefore—efficient therapies are still lacking. The current manuscript focuses on new findings on its pathogenic mechanisms and aims to provide practical therapy recommendations.

## Introduction

Hidradenitis suppurativa (HS) is a chronic skin disorder, which profoundly decrease quality of life because of emotional, physical and psychologic consequences (1–6). This recurrent inflammatory skin condition with an estimated prevalence of 1–4% is more frequent in women (2, 5, 7, 8). Most frequently, the age of onset is between 20 and 40 years of life, although postmenopausal women and prepubertal children with new-onset HS have been reported (2, 5, 9, 10). The clinical manifestations range from inflamed nodules and abscesses to draining sinus tracts and formation of scars (5, 11, 12). The pain, drainage, malodor, and disfigurement associated with HS all contribute to the remarkable psychosocial impact of the disease with negative effects on the patients' quality of life that cannot be overestimated (5, 11, 12). In fact, HS is associated with an increased risk of completed suicide, an increased risk of adverse cardiovascular outcomes and increased all-cause mortality independent of measured confounders (13, 14). Unfortunately, the exact pathogenesis of HS remains unclear. Despite the existence of multiple therapeutic approaches and combinational treatments, effective management of moderate to severe HS remains elusive in many cases. In this manuscript, we summarize the current knowledge about pathophysiology and treatment of HS.

## Pathogenesis

### Mechanism

The precise pathogenesis of HS remains still unclear. This chronic skin disorder might be caused by genetic, endocrine, environmental, and microbiological factors (15). The primary event in disease development is thought to be follicular occlusion, based on histopathological observations in very early lesions (16, 17). Follicular occlusion is likely due to infundibular keratosis and hyperplasia of the follicular epithelium, which leads to accumulation of cellular debris and formation of cysts (16, 18–20). The hair follicle eventually ruptures, followed by release of follicular contents to the dermis, which induces a significant expression of inflammatory mediators and recruitment of inflammatory cells. The end results are abscess formation, painful inflammation and sinus tract formation and scarring, in later stages (16, 18–20).

### Genetics

Two main observations point to a genetic background of HS. First, approximately one third of HS patients have at least one family member also suffering from HS, suggesting an inheritable genetic predisposition (21). Second, families with several mutations or changes in genes of the gamma-secretase family (including nicastrin (NCSTN), presenilin 1 (PSEN1), presenilin enhancer 2 (PSENEN) gene mutation) show an autosomal dominant inheritance of HS, indicating a pathogenic role of these genes in HS (22–26). The resulting alteration in γ-secretase function might result in HS by affecting downstream Notch signaling in the skin (23).

However, these patients show a severe disease phenotype not entirely typical of “sporadic” HS, raising questions as to what degree this genetic background is representative for all cases of HS. Nevertheless, taken together current evidence strongly supports a genetic predisposition for HS development.

### Inflammatory Pathways in Hidradenitis Suppurativa

The excessive inflammatory response seen in lesional skin of HS is thought to be triggered by a combination of genetic, anatomical, immunological, and environmental factors (27, 28). Increased activity of dendritic cells and T cells cause keratinocyte hyperplasia via the actions of IL-23 and IL-12 and a Th17 immune response (27–30). As HS progresses, increased levels of interleukin (IL)-1, tumor necrosis factor (TNF), IL-17, caspase-1, S100A8, S100A9, and IL-10 appear in the tissue together with a recruitment of neutrophils, mast cells and monocytes (16, 27, 28, 31–34). Recent evidence further points to autoinflammatory mechanism in HS. HS skin shows increased formation of neutrophil extracellular traps (NET). Intriguingly, immune responses to neutrophil and NET-related antigens have been linked to enhanced immune dysregulation and inflammation (35). In combination with the strong type I IFN signature in HS skin, these findings suggest a key involvement of the innate immune response in the pathogenesis of HS (36). As healing from the remarkable inflamation progresses, tunneling and scarring of the tissue occur (27, 28, 31). The development of sinus tracts and scarring is associated with matrix metalloproteinase-2 (MMP-2), transforming growth factor beta (TGF-ß) and (Intercellular adhesion molecule-1 (ICAM-1), with possible augmentation of TGF-ß and ICAM-1 signaling via specific components of the microbiome (27, 28).

### Bacteria

Bacterial involvement in HS pathogenesis remains highly debated (37, 38). A number of studies have shown that cultures of HS lesions are predominately sterile or contain only commensal skin flora (38, 39). As a result, primary infection is viewed as an unlikely cause of HS (38, 39). In addition, the lack of strong therapeutic effect of antibiotic treatment further argues against a primary infectious cause (40). However, it is still possible that microbial “dysbiosis” and an altered skin microbiome are important factors contributing to HS pathogenesis. In fact, the cutaneous microbiome in patients with HS is substantially different from that of normal donors. A variety of partly overrepresented, partly unique species have been isolated from HS lesions and linked to various features of the disease (41, 42). Yet, their pathogenetic contribution has remained elusive, in large part because of the technical and experimental difficulties of investigating their causal role in humans (38, 42). Furthermore, the microanatomical intricacies of HS skin with its fistulas and sinus tracts represent an important basis for biofilm formation, bacterial colonization, and secondary infection, which contribute to disease exacerbations, suppuration and extension of lesions (38, 43). Although the role of bacterial biofilm in HS is highly debated, the association between biofilm and the number of regulatory T cells (TREG) in a recent study supports the concept of dysbiosis as a factor in the preclinical HS lesions (44). In summary, microbial colonization and infection are likely pathogenic contributors to HS, but it is still unclear if the colonization of bacteria is a primary or secondary event in the evolution of HS (37). Since the follicular infundibulum is populated by a broad microbiome, deficiencies in the follicular skin immune system might cause microbial overgrowth. However, another theories suggest that an overactive immune system might contribute to a remarkable inflammatory response to harmless, normal flora (16).

### Other Associated Factors

Mechanical stress (45), metabolic syndrome (2, 37, 38), diet (46), smoking (2, 38), and hormonal factors (47) have been reported in previous studies to contribute to the development or exacerbation of HS. While the epidemiological evidence for this is undisputed, at least for some factors such as smoking and obesity, the mechanism by which these factors contribute to HS pathogenesis remain far from clear. Smoking, for instance, is one of the best-documented behavioral risk factors for HS, but the mechanisms by which it triggers HS is poorly understood. There are thousands of chemicals in tobacco smoke, which have the potential to activate keratinocytes, fibroblasts, and immune cells. Mechanistically, these substances may trigger skin cells via two types of receptors: aryl hydrocarbon receptors and nicotinic acetylcholine receptors (16, 48, 49). In keratinocytes, activation of these receptors can lead to infundibular epithelial hyperplasia, acanthosis and excessive cornification (16, 49). Furthermore, tobacco smoke can induce expression of proinflammatory cytokines such as TNF-a, IL-8, IL-1 α, and IL-1β leading to neutrophil chemotaxis, altered function of TREG, as well as activation of Th17 pathways (16, 27, 50–53). Other important risk factors for HS, obesity and diabetes, can also result in overproduction of TNF-α, IL-1β, and IL-6 through activated macrophages in adipose tissue (27, 51–53). In addition, obesity can worsen HS through increased skin-clothing and skin-skin friction, as mechanical stress can increase follicular occlusion and rupture (16, 45).

Hormones are also thought to be involved in HS pathogenesis. This is primarily based on a number of clinical observations and promising effects of antiandrogen treatments. For instance, some female HS patients observe worsening of disease in conjunction with menstruation or after starting hormonal contraception with androgen effects (e.g., progesterone-containing drugs) (47, 54). Hormones can induce occlusion and plugging of the follicle and increase production and composition of sebum, thereby contributing to the vicious cycle of HS skin inflammation (55, 56).

### Suggested Therapy Approach Based on the Current Knowledge

There is no single efficient therapy for the skin disorder. As a result, clinicians typically should choose from different treatment modalities and often have to combine them to achieve disease control. The choice of treatment depends on the distribution and overall severity of disease, the anatomic location and inflammatory activity of the lesions, the patient's comorbidities, as well as treatment cost and availability (2, 5, 9, 38). In order to assess the severity and extent of hidradenitis suppurativa various scoring systems are in common use: hurley stages, sartorius hidradenitis suppurativa score, hidradenitis suppurativa physician global assessment (HS-PGA) and hidradenitis suppurativa clinical response (HiSCR) (57–59).

**Figure 1:**
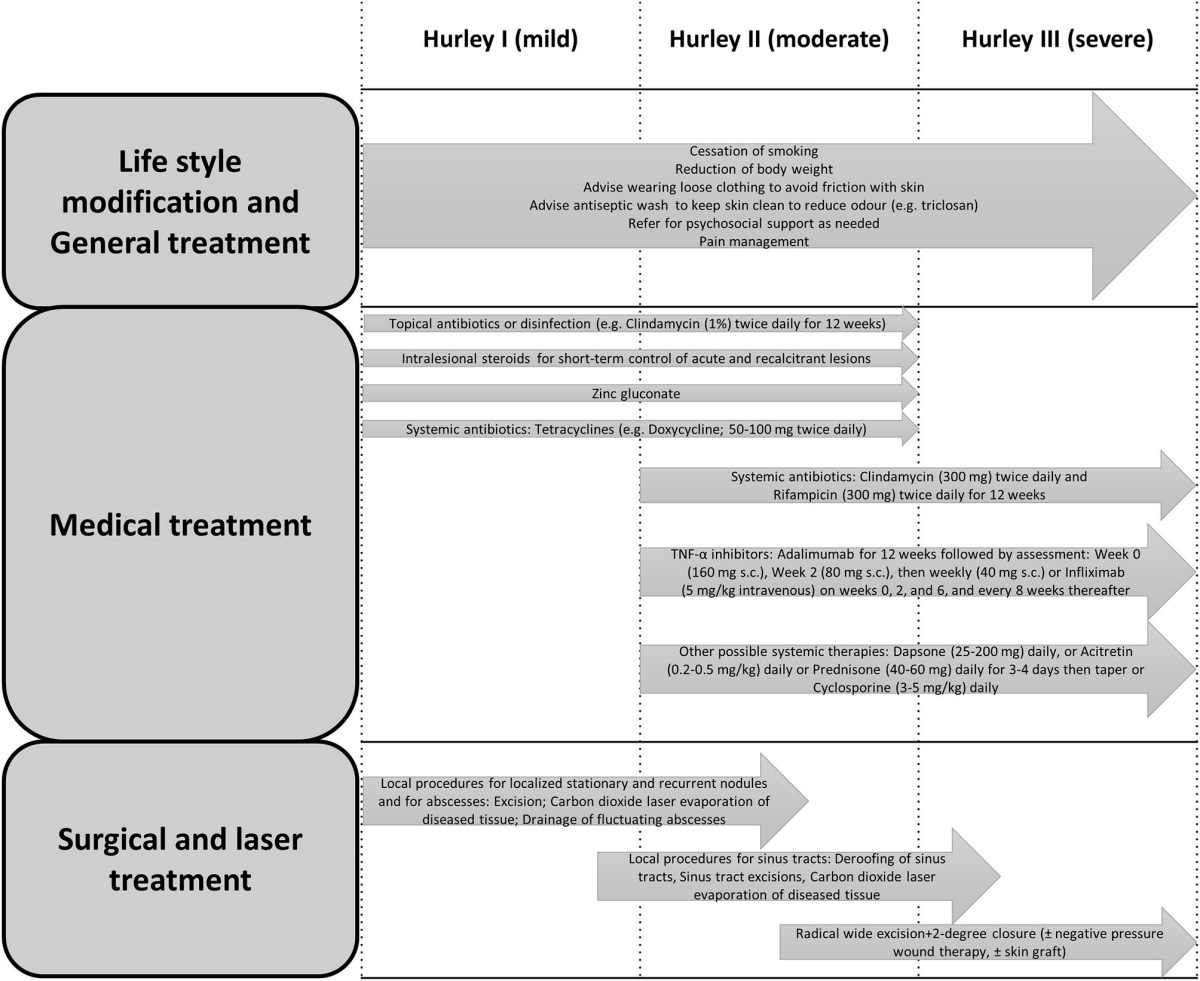
Clinical practice recommendations for the treatment of HS, modified from Hunger et al. and Saunte and Jemec (15, 31).

HS management is often complex. The standard of care management requires an individualized approach in a multifaceted approach, ranging from self-management (e.g., avoidance of skin trauma, pain management, smoking cessation, weight loss and hygiene practices) to local therapies, systemic antibiotics, and a wide range of immunomodulating agents, as well as surgical interventions such as excisional and laser surgeries (6, 15, 38, 39, 60). Additionally, the diagnosis and management of medical comorbidities and referral to psychiatrist might be taken into consideration (61). An overview of a suggested multidimensional approach is summarized in Figure 1.

### Life Style Modification

Because smoking and obesity have the greatest association with the severity of the disease, it is recommended that patients with HS stop smoking and reduce their body weight. In addition, loose-fitting clothing in order is advised to avoid friction and mechanical stress (2, 6, 15). Zinc might be suggested for patients with Hurley stage I-II, as an innate immunity modulator (6, 62).

### Pain Management

Acute and chronic pain is a remarkable contributor to quality of life in individuals with HS, and reduction of inflammation can improve the pain (6, 63). Depression, which plays a crucial role in the perception of pain, is a risk factor for the development of chronic pain in these patients (6, 17, 63). Managing HS pain is complicated. Therefore, a multidisciplinary approach, including pain assessment, pain scoring, and the involvement of a pain specialist, is warranted (6, 17). A variety of alternatives are reported in the recent studies, such as local analgesics, non-steroidal paracetamol, anti-inflammatory drugs, pregabalin, gabapentin, venlafaxine, and duloxetine (17). In more complex cases may require the judicious use of antidepressants (64). In selected patients with severe pain, careful administration of individualized, short-acting opioid analgesics might be necessary (6, 17).

### Local Therapy and Wound Care

Local treatment of this chronic disorder includes antiseptic washes, keratolytic agents and topical antibiotics (6, 60). Selection of dressing is based on the location, skin condition, amount of drainage, cost and patient preference (6, 60). For local treatment of selected large open wounds, use of negative-pressure therapy for a short period of time (1–4 weeks) followed by delayed reconstruction might be effective (6). Clinical experience supported the use of Intralesional corticosteroid injection (e.g., triamcinolone 3–5 mg) only for short-term control of acute and recalcitrant HS lesions (60, 65). Pain is typically reduced fast and a reduction in edema, erythema, suppuration and lesion size occur after a few days. However, the long-term efficacy of this approach remains unclear and local side effects, most notably skin atrophy, have to be carefully monitored (31, 65).

### Antibiotics

Available evidence-based literature on the administration of antibiotics in this disorder is limited and mostly restricted to retrospective studies (66). Determining the frequency and duration of antibiotic use should balance of antibiotic use vs. potential harms associated with antibiotic resistance, because findings of recent studies stated that antibiotic therapy for HS treatment might be inducing antibiotic resistance (67). Typically, HS recurs following cessation of antibiotic therapy, making repeated or long-term treatments necessary, with all their potential harmful side effects (60). For Hurley stage I-II, especially in the absence of abscesses, topical clindamycin 1% is a possible therapy (60, 66, 68, 69). However, it carries a high risk of bacterial resistance (60, 66, 68, 69). If there are frequent exacerbations and/or several lesions the administration of systemic antibiotics can be recommended (66, 69). For example, tetracyclines (e.g., doxycycline 50–100 twice daily) are considered in mild-to-moderate HS for a 12-weeks course or as a long-term maintenance alternative, when appropriate (60). For the patients with Hurley stage II-III systemic clindamycin and rifampicin (300 mg twice daily) could be suggested as a second-line therapy for mild-to-moderate HS or as a first-line or adjunct therapy in the patients with severe disease (60, 66, 70, 71). A triple regimen of moxifloxacin (400 mg once daily), rifampicin (10 mg/kg once daily) and metronidazole (500 mg thrice daily) administered for up to 12 weeks, with metronidazole discontinuation after 6 weeks, might be considered as second- or third-line alternative therapy in moderate-to-severe disease (60, 66). Dapsone might be recommended as an effective and safe alternative therapeutic option for a minority of patients as long-term maintenance therapy (60, 72, 73). The effect could be due to either its anti-inflammatory or antibacterial effects, or both. However, rapid recurrence after stopping the therapy suggests that its anti-inflammatory effects are possible more prominent (60, 72).

### Other Systemic Therapies

A short-term systemic corticosteroid therapy could be recommended for acute conditions or to bridge to other treatments (60). However, long-term systemic steroid therapy tapered to the lowest possible dose might be an alternative option for the patients with severe disease, as an adjunct therapy in patients without sufficient response to standard recommended therapies (60). Hormonal agents, such as estrogen-containing combined oral contraceptives cyproterone acetate, spironolactone, metformin, and finasteride, might be recommended in the female patients, either as monotherapy for mild-to-moderate disease or in combination with other treatments for severe HS (60, 74–77). Isotretinoin could be recommended only as a second- or third-line treatment or in patients with concomitant acne (60, 78, 79). Cyclosporine might be considered in cases of moderate-to-severe disease who have failed or are not suitable for other therapies (60, 80). Recent studies do not recommend the administration of azathioprine or methotrexate in the treatment of HS (60, 81, 82).

### Administration of Biologics in HS

Immunomodulation is becoming popular for moderate-to-severe form of the disease. Targeting the tumor necrosis factor (TNF), interleukin 1 (IL-1), IL-12, and IL-23 has been considered as potential therapies (60, 83).

Adalimumab is recommended as the first-line biologic therapy for moderate-to-severe HS (60, 66), followed by Infliximab and anakinra as second- and third-line options, respectively (66, 84, 85). The recommended dose of adalimumab in HS is 160 mg on Week 0, 80 mg on Week 2 and then 40 mg weekly (60, 66). Recent studies suggest that those patients who do not respond to therapy within 12 weeks (<25% improvement in inflammatory nodules and abscesses) should discontinue the drug (66, 86–89). However, the patients with partial or good should continue the therapy with ongoing assessment (66, 86–89).

Evidence is lacking for other biologic therapies, and any clinical decisions should be based on close monitoring and risk: benefit assessments (66). Recent studies showed administration of other biologics such as other TNF-alpha blockers (66, 90–92), ustekinumab, (93–96) secukinumab, (97, 98) bimekizumab, (99) canakinumab, (3, 100) apremilast, (1, 101) anakinra, (102) guselkumab (103, 104), and rituximab (105) in management of HS.

Most biologics are well-tolerated and show a favorable safety profile in the short- to medium-term. However, long-term safety concerns, including infection risks, the development of malignancy and demyelinating disorders should be to assessed (19). These issues are remarkable in the treatment of HS, where the biologics dosing is generally higher in comparison to other inflammatory skin disorders such as psoriasis. As a result, patient selection is crucial, because complete response is not the norm and not all patients tolerate or respond well to these therapies. Critical monitoring of therapy effects and side effects has to be carefully done under treatment to inform continuous risk-benefit assessment. In case of unfavorable outcome of such assessment, other treatment modalities have to be taken into consideration, such as surgery (19).

### Surgical Modalities

Planning of type of surgery and required margins should be cleared in cooperation with the patient and based on the severity of the disease (15, 39, 66). Local excision of single lesions is only suggested in well-circumscribed, localized cases of Hurley I-II (15). For painful abscesses, no medical therapy will be effective (6, 15, 43, 66, 106, 107). Therefore, surgical drainage might be demanded to relieve pain; however, this should not be considered as sole treatment, because recurrence is most of the patients inevitable (6, 15, 43, 66, 106, 107). Extensive excision, electrosurgical excision (with or without reconstruction) CO_2_ laser could be appropriate for chronic lesions to prevent recurrence (5, 6, 66, 108–111). Wound healing following surgery may be through primary closure, delayed primary closure, secondary intention, grafts, flaps, and/or skin substitutes (5, 6, 66, 108–111). Furthermore, a new surgical approach was described recently, the step-by-step surgery, which consists in consecutively removing portions of HS skin with secondary intention healing (112). Recent studies suggest that continuing medical therapy in the perioperative period might be beneficial and reduce risk of postoperative complications (6).

### Laser Therapy

Most of laser therapies have been focused of modification of disease activity in the groin and axillae, because, the destruction of the pilosebaceous apparatus might prevent the extension of disease (17). Neodymium-doped yttrium aluminum garnet (Nd:YAG) laser is applicable for Hurley stage II-III disease (6, 17, 39). Furthermore, CO_2_ laser excision can be considered in patients with Hurley stage II-III with fibrotic sinus tracts (6).

## Conclusions

In conclusion, HS is characterized by a pathological reaction pattern of the follicular epithelium of the apocrine gland-bearing skin to yet ill-defined insults. As a result, the follicular epithelium becomes hyperplastic, showing hyperkeratosis of the infundibulum, follicular occlusion, and cyst formation. Rupture of such cysts and consecutive invasion of follicular material into the dermis is likely a key step in triggering a remarkable immune response, which lies at the heart of painful inflammation and abscess formation. In later stages, uncontrolled inflammation leads to sinus tract formation and scarring. There is no uniformly effective therapy for HS. As a result, clinicians should take a multifaceted approach depending on the patients' needs and on features of the disease. Therapy modalities range from self-management and topical treatments to systemic antibiotics, immunomodulating agents, as well as surgical interventions. All in all, it is recommended to avoid the trigger factors such as: smoking, obesity, mechanical friction and shaving. Furthermore, local disinfectants and/or topical antibiotics are suggested in order avoid bacterial superinfections and reduce inflammation. If topical agents are not sufficient, systemic antibiotics are typically given. If antibiotics are not sufficient or no longer effective, other systemic therapies (e.g., adalimumab, dapsone, systemic steroids, or cyclosporine) might be used in patients with refractory disease. Additionally, if needed different surgical interventions might be taken into consideration in cooperation with the patient and based on the severity of the disease.

## Author Contributions

SS, RH, and CS designed the study and performed acquisition, analysis, interpretation of data, and wrote the manuscript, and performed critical revision of the manuscript for important intellectual content.

### Conflict of Interest

The authors declare that the research was conducted in the absence of any commercial or financial relationships that could be construed as a potential conflict of interest.
